# Surgical Cases (?)

**Published:** 1893-01

**Authors:** J. A. Tomhagen

**Affiliations:** Philadelphia, Pa.


### SURGICAL CASES. (?)

J. A. Tomhagen, M. D., Philadelphia, Pa.

January 19th, 1892.—F., aet. ten, was struck with a stone on back of head, about one inch upward and backward from mastoid process, three weeks prior to present illness. Dr. Medley being in the vicinity during my absence, was called in to prescribe for the boy. The Doctor found him stupid, with besotted appearance and sluggish pupils, high fever, and rapid pulse. The tout-ensemble led her to exhibit Arnica$^{cm}$.

January 20th.—I found him with temperature 106 and pulse 160; very stupid. When I called his name aloud he would open his eyes and then relapse into stupor. The mother informed me that he had had an *involuntary* stool during the night, and had frequently *jumped up in bed and looked down where he had been lying, as though he saw something of which he was afraid, then he would lie down, and immediately* lapse into stupor.

I regarded the *involuntary* stool as pathogenetic, since it had not existed before, and is very characteristic of the remedy. Continue medicine.

January 21st.—Sleep more restful and no *involuntary* stool. Temperature 105. Improvement continues from this time on.

February 2d.—Head sweats so he wets pillows. Calc.$^{200}$ controlled this. In eleven days the patient was discharged, cured. One dose of Arn. accomplished the work. The *involuntary* stool, *coming as it did*, was a warning to me to let the remedy alone.

Case 2. June 15th, 1885.—Mr. E. L. B., forty, consulted me for an *ulcer* on right side of tongue near anterior portion, it was as large as a dime, raw looking, but painless, and surrounding mucous membrane appeared as if bleached. This *ulcer* worried him, and had been the point of attack for ten years off and on for antiquated doctors. It was difficult to induce him to divulge any symptoms, for he was preoccupied with the ulcer, and would constantly revert to it, saying, "he would be *well* if that were healed."

I noticed that both commisures of his mouth were sore when he twirled his mustache about his fingers. On calling his attention to the *fact* he incidentally remarked that that always occurred when he had dyspepsia. Furthermore, I learned that he cared for nothing but *pickles* when he had these attacks of indigestion, and was usually constipated. Ant-crud.$^{3m}$.

January 25th.—Has a urethral discharge, "the likes of which" he had not had for twenty years. He desired to know how that could come about without having been exposed. On carefully questioning him I learned that he had had gonorrhœa several times in his youth, and invariably succeeded in curing (?) it with injections. C. M.

July 12th.—Discharge persists and is bland and thick yellowish green. Ulcer on tongue getting smaller and sores in corners of mouth almost gone; appetite better. C. M.

July 19th.—Improving in every respect; ulcer smaller and sores about mouth healed; discharge from urethra same. C. M.

August 10th.—Ulcer well, but discharges somewhat. Puls.$^{2m}$.

August 25th.—Discharge almost gone. C. M.

September 2d.—Discharged cured.

### Remarks.

This proves to me again how beautifully a case unfolds under the benign influence of the appropriate medicine.

The *ulcer* had been treated locally, exclusively, prior to the administration of Ant-crud., with unsatisfactory results.

The cause undoubtedly was suppressed gonorrhœa, since the ulcer and concomitant symptoms gradually vanished when the discharge supervened.

The ulcer, *per se*, was ignored in selecting the remedy, and those symptoms, generally considered accidental, were regarded of prime importance.

One dose of Ant-crud. was sufficient to re-establish a discharge that had been latent, as it were, over twenty years. Puls. having the same characteristic discharge and desire for *sour* things, and following Ant-crud. well, was given to finish the cure.

Homœopathy is simple, exact, and pre-eminently satisfactory.

Adjourned to four P. M.

